# Protective Effects of Lanostane Triterpenoids from Chaga Mushroom in Human Keratinocytes, HaCaT Cells, against Inflammatory and Oxidative Stresses

**DOI:** 10.3390/ijms241612803

**Published:** 2023-08-15

**Authors:** Jihyun Park, Thi Minh Nguyet Nguyen, Hyun-ah Park, My Tuyen Thi Nguyen, Nan-young Lee, So-young Ban, Kyu-been Park, Chang-kyu Lee, Jaehan Kim, Jong-Tae Park

**Affiliations:** 1 CARBOEXPERT Inc., Daejeon 34134, Republic of Korea; jhpark@carboexpert.com (J.P.); minhnguyet.usth@gmail.com (T.M.N.N.); hapark@carboexpert.com (H.-a.P.); nylee@carboexpert.com (N.-y.L.); syban@carboexpert.com (S.-y.B.); kbpark@carboexpert.com (K.-b.P.); cklee@carboexpert.com (C.-k.L.); 2 Department of Food and Nutrition, Chungnam National University, Daejeon 34134, Republic of Korea; mytuyen1108@gmail.com (M.T.T.N.); jaykim@cnu.ac.kr (J.K.); 3 College of Agriculture, Can Tho University, Can Tho City 94115, Vietnam; 4 Department of Food Science and Technology, Chungnam National University, Daejeon 34134, Republic of Korea

**Keywords:** lanostane triterpenoid, inotodiol, keratinocyte, cellular senescence, anti-inflammation

## Abstract

Inotodiol, a lanostane-type triterpenoid, and many phytochemicals from Chaga mushrooms have been investigated for various allergic diseases. However, the anti-aging and anti-inflammatory activities of inotodiol under different types of oxidative stress and the impact of inotodiol on collagen and hyaluronan synthesis have not been sufficiently studied. Lanostane triterpenoids-rich concentrate, which contained 10% inotodiol as major (inotodiol concentrate), was prepared from Chaga and compared with pure inotodiol in terms of anti-inflammatory activities on a human keratinocyte cell line, HaCaT cells, under various stimulations such as stimulation with ultraviolet (UV) B or tumor necrosis factor (TNF)-α. In stimulation with TNF-α, interleukin (IL)-1β, IL-6, and IL-8 genes were significantly repressed by 0.44~4.0 μg/mL of pure inotodiol. UVB irradiation induced the overexpression of pro-inflammatory cytokines, but those genes were significantly suppressed by pure inotodiol or inotodiol concentrate. Moreover, pure inotodiol/inotodiol concentrate could also modulate the synthesis of collagen and hyaluronic acid by controlling COL1A2 and HAS2/3 expression, which implies a crucial role for pure inotodiol/inotodiol concentrate in the prevention of skin aging. These results illuminate the anti-inflammatory and anti-aging effects of pure inotodiol/inotodiol concentrate, and it is highly conceivable that pure inotodiol and inotodiol concentrate could be promising natural bioactive substances to be incorporated in therapeutic and beautifying applications.

## 1. Introduction

Human skin is a complex and typically specialized organ, the largest in the human body, with a multifaceted structure that has crucial roles in maintaining one’s health. The multiple layers of skin protect us from external stressors, including harmful radiation from the sun, chemicals, and pathogens. In addition, skin plays an important role in temperature regulation, sensation, and even immune surveillance. Three of the critical cells involved in the formation and function of the skin are keratinocytes, fibroblasts, and melanocytes. Keratinocytes are the primary cell type in the epidermis, the outermost layer of the skin, constituting about 90% of its cells. Keratinocytes originate from basal cells in the basal layer of the epidermis. They undergo a systematic process of differentiation as they migrate from the basal layer to the stratum corneum, the outermost layer of the skin [1,2,3].

Fibroblasts are the most abundant cells in the dermis, the layer of skin beneath the epidermis. They play a crucial role in producing the extracellular matrix (ECM), the structural framework for the skin. The ECM is composed of collagen and elastin, proteins that provide strength and elasticity, respectively, to the skin. Melanocytes, which have different roles from the other skin cells, are located in the basal layer of the epidermis and are responsible for producing melanin, the pigment that gives skin, hair, and eyes their color. Even though pigment generation is not favored for skin beauty, melanin plays a crucial role in protecting the skin from harmful ultraviolet (UV) radiation [3,4].

Pro-inflammatory cytokines, one of the inflammatory mediators, are produced by multiple types of cells such as macrophages, mast cells, lymphocytes, and keratinocytes and contribute to the initiation of various diseases, including vascular disease, lipid metabolism, cancer, and aging-related diseases [1,2]. Keratinocytes, also well known for the synthesis of keratin, produce proinflammatory cytokines, such as tumor necrosis factor (TNF)-α and interleukin (IL)-1β, when they detect antigen and play a crucial role in the immune response in the skin [3]. Pro-inflammatory cytokines are also induced by ultraviolet (UV) radiation for skin defense responses [4]. Even though these cytokines are important for modulation of the immune response for protection of the host, excessive production of pro-inflammatory cytokines may cause chronic inflammation and negative consequences [5]. 

Inflammaging, which was coined in 2000 by Professor Claudio Franceschi, is a concept that suggests that low-grade and persistent proinflammatory status, which is characterized by high levels of pro-inflammatory markers, might lead to long-term aging progression and tissue damage [4,6,7,8]. If inflammaging occurs in the skin, it can cause cancer or skin diseases related to aging [9]. It is not only aging that causes inflammaging, but also external factors such as UV radiation or smoking can be reasons for inflammaging [10]. In more details, external stressors, including UV, microbiome, and particulate matter that come from air pollution, can induce various pro-inflammatory signals through aryl hydrocarbon receptors, mitogen-activated protein kinases (MAPK)-activator protein (AP)-1, and nuclear factor kappa-light chain-enhancer of activated B cells (NF-kB). In addition, cellular senescence of keratinocytes, fibroblasts, and melanocytes in the skin can cause the senescence of skin-resident immune cells like Langerhans cells, dendritic cells, macrophages, and T cells. Taken together, imbalance or loss of integrity in the skin significantly mitigates not only skin health but also the overall health status [7,8,9]. 

Chaga mushroom is a well-known source of bioactive triterpenes and has been used as a traditional medicinal mushroom in various regions of the world. Inotodiol, a lanostane-type triterpenoid from the Chaga mushroom, has been used as a traditional medicine for diabetes, cardiovascular disorders, and tuberculosis [11,12]. Numerous studies have investigated inotodiol’s biological activities, and it has been proven to have antitumor, anti-inflammatory, antiallergic, antiviral, antiaging, and antioxidant properties [13,14,15,16]. Recently, there has been a movement to identify novel, safe, and effective molecules that can be used for the treatment of various diseases, including inflammatory diseases. So far, it has been reported that inotodiol exerts anti-inflammatory activity without showing detrimental effects in in vivo and in vitro studies [17]. Also, inotodiol shows an anti-aging effect under oxidative stress by inhibiting MAPK-NOX5 and NF-κB signaling pathways [16]. However, the anti-aging and anti-inflammatory activities of inotodiol under different types of oxidative stress and the impact of inotodiol on collagen and hyaluronan synthesis have not been sufficiently studied. We therefore complemented our previous data in terms of the anti-aging and anti-inflammatory effects of inotodiol.

The aims of this study were (1) to evaluate the impact of inotodiol on pro-inflammatory cytokine production by keratinocytes stimulated with UV or TNF-α and (2) to assess how inotodiol affects hyaluronic acid synthesis in HaCaT cells in the presence of TNF-α or not. Here, we demonstrated for the first time that inotodiol is capable of reducing inflammation induced by UVB and TNF-α and upregulates hyaluronan and collagen synthesis-related genes in HaCaT cells, which imply a potential role for inotodiol in mitigating or delaying inflammaging.

## 2. Results

### 2.1. Determination of Major Triterpenes in the Inotodiol Concentrate

**Figure 1:**
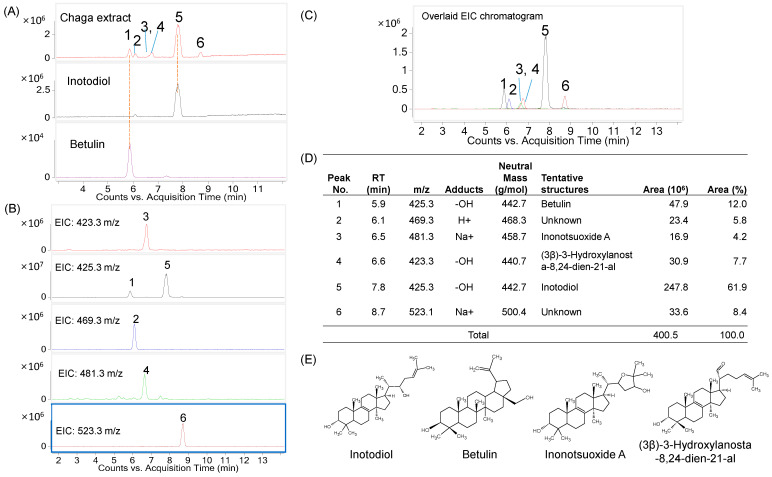
Composition of triterpenes in the inotodiol concentrate produced from Chaga mushroom extract. (**A**) TIC chromatogram of the inotodiol concentrate. Each peak is numbered by its retention time and consistently used through (**A**–**D**); (**B**) extract ion chromatogram; (**C**) overlaid chromatogram; (**D**) table of abundance of triterpene compositions; (**E**) structures of inotodiol and other triterpenes.

The inotodiol concentrate was subjected to analysis using LC/MS in positive mode to determine its triterpene composition. Six compounds were found in the product (Figure 1). These compounds exhibited mass-to-charge ratios (*m*/*z*) of 423.3, 425.3 (two compounds), 469.3, 481.3, and 523.1 *m*/*z*. Notably, compounds 1 and 5 shared the same *m*/*z* value of 425.3 [M-OH]^+^. The retention times (RT) of peaks 1 and 3 were 5.9 and 7.8 min, respectively. They corresponded to the standards of inotodiol and betulin, as shown in Figure 1A. Peak 3, with an *m*/*z* value of 481.3 [M + Na]^+^, could be identified as inonotsuoxide A. Furthermore, peak 4 exhibited an *m*/*z* value of 423.3 [M-OH]^+^ and could be (3β)-3-hydroxylanosta-8,24-dien-21-al. Supplementary Figure S1 illustrates the mass spectra of the major compounds present in the inotodiol concentrate.

Based on the analysis of peak areas, inotodiol was found to be the most abundant lanostane triterpenoid, accounting for approximately 69.1% of the total area. Betulin followed as the second most abundant compound, comprising approximately 12% of the total peak area. The remaining compounds exhibited peak intensities of less than 10% of the total intensity.

### 2.2. Evaluation of Cytotoxic Effect of Inotodiol and Inotodiol Concentrate

**Figure 2:**
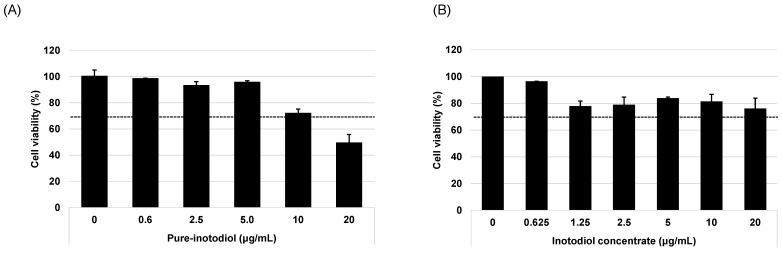
Effect of pure inotodiol and inotodiol concentrate on cell viability in HaCaT cells. Cytotoxicity (MTT assay) of pure-inotodiol (**A**) and inotodiol concentrate (**B**).

The MTT assay was used to evaluate the cytotoxicity of pure inotodiol and inotodiol concentrate using the HaCaT cell line, and cytotoxicity was determined as per ISO EN 10993-5 criteria. Cell viability under 70% of the vehicle-treated sample (0 μg/mL) was considered to be toxic [18]. Cells were treated with various concentrations of inotodiol/inotodiol concentrate (0~20 μg/mL) for 24 h. Inotodiol was non-toxic (viability was >70%) other than at the highest concentration of 20 μg/mL. Inotodiol concentrate was non-toxic at all tested concentrations (viability was >70%). At a lower concentration (~5 μg/mL), inotodiol shows more than 90% cell viability, but it gradually decreased with the increase in concentrations of inotodiol (Figure 2). In inotodiol concentrate, no concentration-dependent decrease in terms of cell viability was observed (Figure 2B). According to the results for cell viability, 5 μg/mL of inotodiol and 20 μg/mL of inotodiol concentrate were chosen as the highest concentrations for in vitro cell line assays.

### 2.3. Impact of Inotodiol and Inotodiol Concentrate on Pro-Inflammatory Cytokine Expression after Treatment with TNF-α in HaCaT Cells

**Figure 3:**
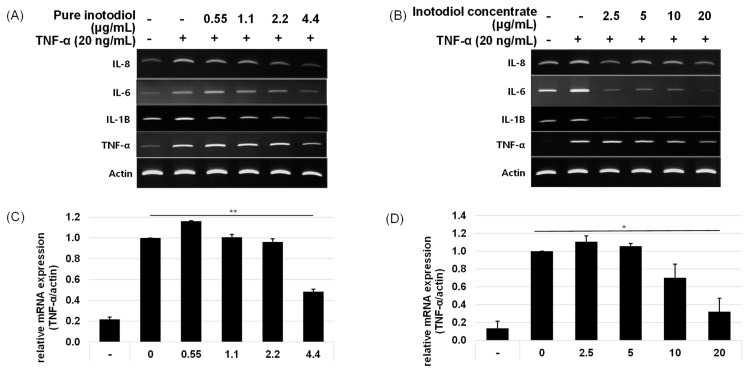
Anti-inflammatory effects of pure inotodiol (**A**,**C**) and inotodiol concentrate (**B**,**D**) on HaCaT cells treated with TNF-α (20 ng/mL). Statistical significance was denoted as * for *p* < 0.05 and ** for *p* < 0.01.

To examine the effect of inotodiol/inotodiol concentrate on the pro-inflammatory cytokine mRNA expression level after treatment with TNF-α in HaCaT cells, the mRNA expression of IL-1β, IL-6, and IL-8 were evaluated by real-time qPCR. The mRNA expression levels of IL-1β, IL-6, and IL-8 increased by treatment with TNF-α (Figure 3).

**Figure 4:**
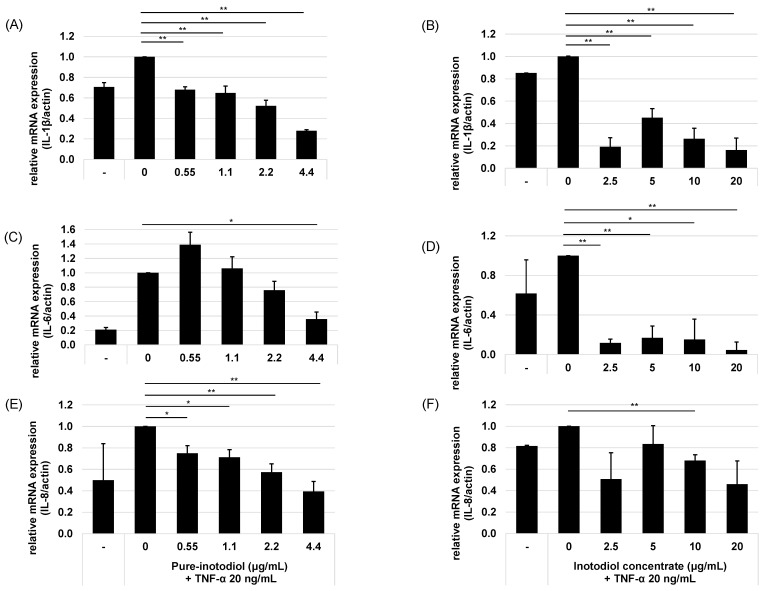
Effects of pure inotodiol (**A**,**C**,**E**) and the inotodiol concentrate (**B**,**D**,**F**) on expression in proinflammatory cytokines of HaCaT cells treated with TNF-α (20 ng/mL). Statistical significance was denoted as * for *p* < 0.05 and ** for *p* < 0.01.

The addition of inotodiol/inotodiol concentrate, however, significantly reduced the mRNA expression level of these cytokines, especially at higher concentrations. The addition of inotodiol in TNF-α presence reduced the mRNA expression level of these cytokines in a dose-dependent manner (Figure 4). Cells treated with inotodiol concentrate showed decreased mRNA expression of the cytokines at the lowest tested concentration (2.5 μg/mL).

### 2.4. Impact of Inotodiol and Inotodiol Concentrate on Pro-Inflammatory Cytokine Expression after Treatment with UV in HaCaT Cells

**Figure 5:**
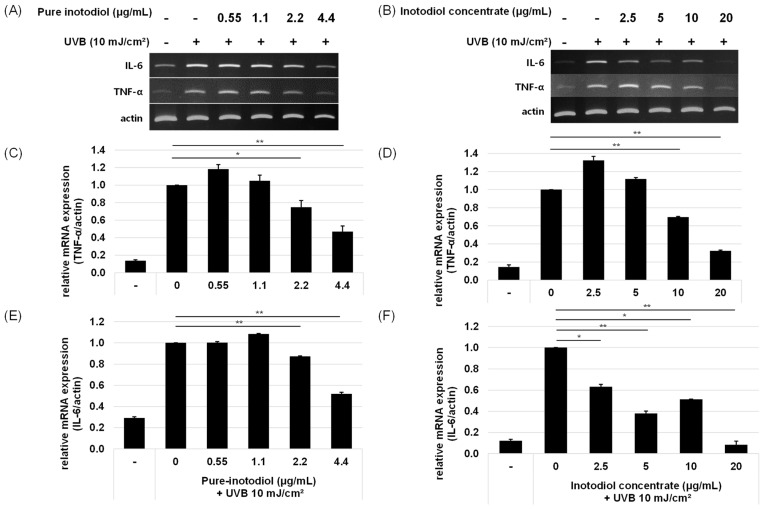
Effects of pure inotodiol (**A**,**C**,**E**) and the inotodiol concentrate (**B**,**D**,**F**) on expression of pro-inflammatory cytokines in HaCaT cells induced by UVB exposure (10 mJ/cm^2^). Statistical significance was denoted as * for *p* < 0.05 and ** for *p* < 0.01.

Inflammation is one of the important mediators of photoaging, and UV radiation promotes the release of inflammatory mediators such as IL-1 and IL-6 through the induction of pro-inflammatory genes in keratinocytes [19]. To investigate the ability of inotodiol/inotodiol concentrate to reduce inflammation levels, the mRNA expression levels of two pro-inflammatory cytokines, IL-6 and TNF-α were assessed. By addition of inotodiol/inotodiol concentrate, the mRNA expression level of IL-6 and TNF-α decreased (Figure 5). Especially above 2.2 μg/mL inotodiol, reduced cytokine expressions. In cells treated with inotodiol concentrate, the IL-6 mRNA expression level at the highest concentration (20 μg/mL) returned almost to the basal level.

### 2.5. Impact of Inotodiol/Inotodiol Concentrate on Hyaluronan Synthesis and Collagen

**Figure 6:**
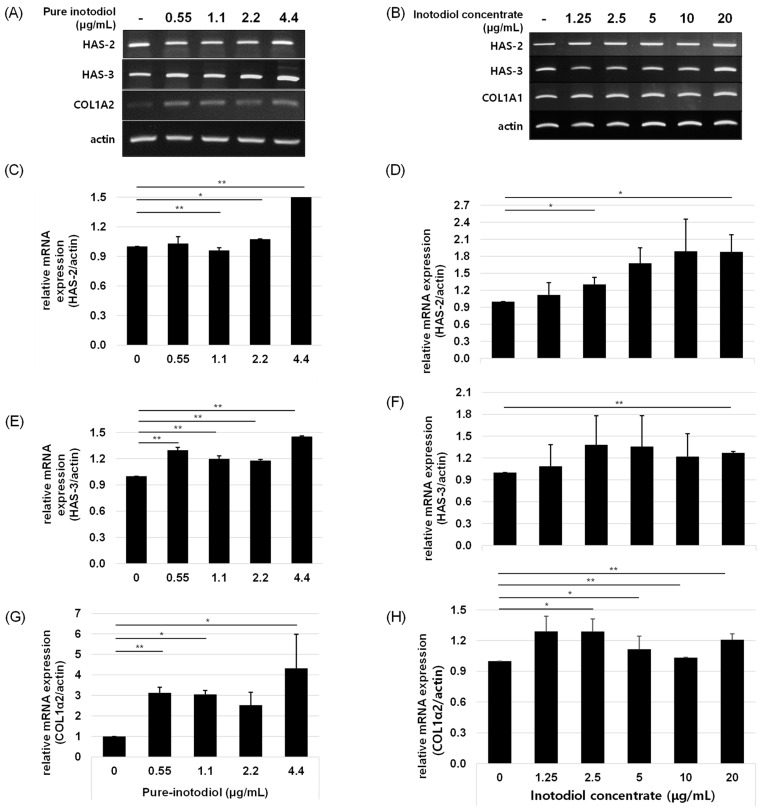
Effects of pure inotodiol (**A**,**C**,**E**,**G**) and the inotodiol concentrate (**B**,**D**,**F**,**H**) on expression of genes involved in collagen (COL1α2) and hyaluronan (HAS-2 and HAS-3) synthesis in HaCaT cells. Statistical significance was denoted as * for *p* < 0.05 and ** for *p* < 0.01.

To investigate if inotodiol/inotodiol concentrate affects collagen synthesis, HaCaT cells were treated with inotodiol/inotodiol concentrate, and the mRNA expression level of COL1A2 was determined by qRT-PCR. Treatment with inotodiol/inotodiol concentrate increased COL1A2 expression in HaCaT cells. In addition, to examine the role of inotodiol/inotodiol concentrate in hyaluronic acid expression, HAS2 and HAS3 mRNA expression levels were also assessed by qRT-PCR. Hyaluronic acid (HA) synthesis is regulated by an enzyme called hyaluronic acid synthases (HAS) [20], and three isoforms of HAS, HAS1, HAS2, and HAS3, have been reported so far [1,21]. As shown in Figure 6, inotodiol/inotodiol concentrate increased HAS2 and HAS3 expression. 

**Figure 7:**
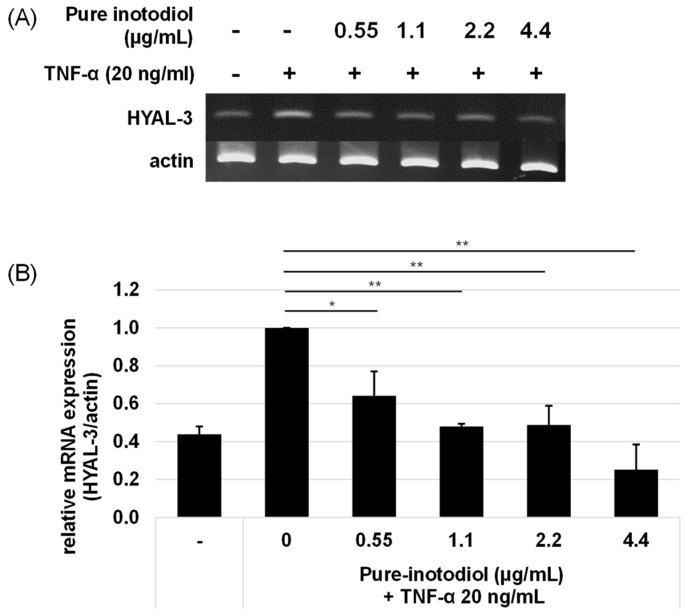
Effects of pure inotodiol on expression of genes involved in hyaluronan (HYAL-3) degradation in HaCaT. Agarose gel analyses of the HYAL-3 PCR product (**A**) and their quantification results (**B**) are shown. Pro-inflammatory TNF-α was used for inducing inflammation in HaCaT cells. Statistical significance was denoted as * for *p* < 0.05 and ** for *p* < 0.01.

Hyaluronidase (HYAL) has HA degrading activity through cleavage at the β-(1,4)-linkage [22]. To investigate the influence of inotodiol on expression of HYAL 3 under stimulation with TNF-α (20 ng/mL), HaCaT cells were incubated with various concentrations of inotodiol under TNF-α stimulation for 6 h. By adding TNF-α to HaCaT cells without inotodiol, HYAL-3 mRNA expression increased (Figure 7). Treatment with inotodiol, however, significantly reduced HYAL-3 mRNA expression even at low concentrations (0.55 μg/mL).

## 3. Discussion

Multiple stresses or stressors, such as oxidative, chemical/physical, or antigenic stress, lead to constant stimulation of the immune response, which can be characterized as inflammaging [6]. In the skin, tissue damage or other skin diseases can be triggered by inflammaging. Cellular senescence in the skin includes the loss of functions and inflammatory responses of keratinocytes, fibroblasts, and skin-resident immune cells [23]. Cross talk, triggered by chronic inflammation in the skin, between immune cells and skin cells probably makes the situation worse [9]. Inotodiol has been studied for various biological activities, and its anti-allergic and anti-inflammatory functions are its well-known activities [24,25,26,27,28,29]. UV irradiation causes acute inflammation that is induced by oxidative stress [30]. UV irradiation induces the accumulation of ROS and results in the activation of NF-κB, which can upregulate expression of genes involved in inflammation [31].

In this study, we investigated the functions of inotodiol/inotodiol concentrate in terms of modulation of UV- or TNF-α-induced inflammation for the first time. Previously, it has been reported that inotodiol has anti-oxidative and anti-allergic activities in vivo and in vitro [32,33,34]. Moreover, recent study shows that inotodiol has anti-aging effects by protecting human dermal fibroblasts by blocking MAPK-NOX5 and NF-κB activation and suppressing aging gene expression [16]. However, it is still not elucidated if inotodiol affects cytokine expression after stimulation with UV and TNF-α and how inotodiol impacts extracellular matrix production after stress stimulation. Here, we demonstrated that inotodiol reduced UV- and TNF-α-induced pro-inflammatory cytokine expressions and upregulated hyaluronan synthesis by increasing HAS2 and HAS3 expression, which results in augmentation of HA synthesis. In addition, inotodiol prevented degradation of HA by downregulating hyaluronidase-3 expression under TNF-α stimulation. Col1α2, which has an important role in collagen synthesis, was up-regulated, too. Inotodiol concentrate showed similar biological activity patterns, but its potency was significantly lower than that of inotodiol. Taken together, the results demonstrated potent anti-inflammatory and anti-aging activities of inotodiol in skin cells and imply a potential use of inotodiol in the cosmetic industry. 

Intrinsic aging and extrinsic aging trigger skin aging through a complex biological process [34]. Ultraviolet (UV) radiation is one of the external factors that causes extrinsic aging, also called photoaging. In HaCaT cells that were exposed to UV or TNF-α, expression of pro-inflammatory cytokines such as IL-1β, IL-6, and IL-8 increased, which verifies that those stress stimuli play crucial roles in terms of skin aging (Figure 3, Figure 4 and Figure 5). TNF-α has been known as a key mediator of immune response and inflammation in the skin through activation of NF-κB signaling [35]. In skin, keratinocytes, epidermal Langerhans cells (LC), and macrophages are major cells that secrete TNF-α, and this TNF-α acts on these cells via autocrine manner and induces other pro-inflammatory cytokines [36]. 

To assess the ability of inotodiol/inotodiol concentrate to modulate inflammation, pro-inflammatory cytokine expressions were measured in HaCaT cells treated with or without inotodiol/inotodiol concentrate under UV or TNF-α stimulation. Inotodiol/inotodiol concentrate protected cells from an increase in pro-inflammatory cytokines against UV or TNF-α stimulation, and these results indicate a positive control of inotodiol/inotodiol concentrate in terms of inflammaging. Regarding control of IL-1β expression by treatment with inotodiol/inotodiol concentrate, expression of IL-1β decreased even with low concentrations of inotodiol/inotodiol concentrate. It has been reported that pro-inflammatory cytokines, including IL-1β promote collagen degradation and downregulate collagen production [37]. In our study, we demonstrated that the addition of inotodiol/inotodiol concentrate to HaCaT cells increased COL1A2, which means inotodiol/inotodiol concentrate positively modulates HaCaT cells in terms of collagen synthesis. Moreover, inotodiol up-regulated HAS2 and HAS3, which indicates the ability of inotodiol/inotodiol concentrate to increase hyaluronic acid expression, which is important to protect skin from senescence [35]. We could not determine all the biologically active components in inotodiol concentrate. Therefore, there could be other potent functional compounds in the product. Nevertheless, our findings suggest that inotodiol/inotodiol concentrate effectively control skin aging via suppression of pro-inflammatory cytokines and augment the skin’s ability to maintain collagen and hyaluronic acid synthesis. 

## 4. Materials and Methods

### 4.1. Materials

Dulbecco’s Modified Eagle’s medium (DMEM), Dulbecco’s Phosphate Buffered Saline (DPBS), fetal bovine serum (FBS), and penicillin-streptomycin solutions (P/S) were purchased from Sigma-Aldrich (St. Louis, MO, USA). 3-(4,5-dimethylthiazol-2-yl)-2,5-diphenyltetrazolium bromide (MTT) was purchased from Thermo Scientific (Waltham, MA, USA), and both AccuPower CycleScript RT premix and AccuPower Taq PCR Premix primers were purchased from Bioneer Corp. (Daejeon, Republic of Korea).

### 4.2. Preparation of Inotodiol Concentrate and High Purity Inotodiol

Dried Chaga mushrooms were purchased from Jungwoodang (Seoul, Republic of Korea). Chaga mushroom powder (200 g) was treated with 2 L of 70% ethanol and stirred at 50 °C for 4 h. After centrifugation (12,000× *g*, 10 min) at room temperature, the supernatant was obtained and concentrated using a vacuum rotary evaporator (Deahan Science Co., Daejeon, Republic of Korea) to a final volume of 200 mL. The concentrate was kept at 4 °C for 15 h, and the precipitant was separated by centrifugation (10,000× *g*, 10 min). The separated precipitant was freeze-dried and kept in an airtight bottle at 4 °C before use. The product obtained after freeze-drying was designated as inotodiol concentrate, which had approximately 10% inotodiol. To obtain high-purity inotodiol, inotodiol concentrate was further purified using a recycling HPLC system (MPLC, YMCKOREA, Seongnam, Republic of Korea) equipped with reverse-phase chromatography (ODS-AQ, 50 × 500 mm, YMCKOREA) [14]. Finally, 95% pure inotodiol was produced and used for the in vitro assays.

### 4.3. HPLC-MS/MS Analyses of Inotodiol Concentrate and High Purity Inotodiol

The triterpene compositions were identified using Agilent-1290 UPLC/6470A and Agilent-1290 UPLC/6430A QQQ system (Agilent Technologies Korea, Seoul, Republic of Korea) equipped with an electrospray ionization (ESI) source. The mobile phase consisted of solutions A (distilled water) and B (methanol), each containing 5 mM ammonium acetate and 0.1% formic acid. The separation was performed on a Zorbax Eclipse Plus C18 column (2.1 × 50 mm, 3.5 µm) (Agilent Technologies, Palo Alto, CA, USA). The solution B gradient was 75% at 0–2 min, 90% at 7 min, 100% at 18–25 min, and 75% at 26–35 min. The injection volume was 2 μL, and the flow rate was set at 0.2 mL/min with a column temperature of 40 °C. The mass spectrometer was performed in positive mode with the following parameters: Dry gas temperature: 270 °C; Dry gas flow rate: 10 L/min; Nebulizer pressure: 40 psi; Sheath gas temperature: 300 °C; Sheath gas flow rate: 11 L/min; Capillary voltage: 3500 V (positive); Nozzle voltage: 500 V (positive). The triterpene composition was identified using the scan mode with a mass range from 300 to 1000 *m*/*z*.

### 4.4. Cell Culture

HaCaT cells, a human epithelial keratinocyte cell line, were kindly provided by Dr. Jongil Park from Chungnam University Medical School. HaCaT cells were cultured in Dulbecco’s Modified Eagle’s Medium supplemented with 10% fetal bovine serum and 1× penicillin (100 units/mL)-streptomycin (100 μg/mL) at 37 °C under 5% CO_2_.

### 4.5. Cell Viability Assay

A standard MTT assay was performed to determine cytotoxicity according to the manufacturer’s protocol (M6494, Invitrogen, Camarillo, CA, USA) [16]. HaCaT cells were seeded at a density of 1 × 10^5^ cells/well and incubated for 24 h. Cells were treated with various concentrations (0.6~20 μg/mL) of pure inotodiol and inotodiol concentrate in a 96-well plate. After treatment with pure inotodiol and inotodiol concentrate for 24 h under 5% CO_2_, the cells were treated with 3-(4,5-dimethylthiazol-2-yl)-2,5-diphenyltetrazolium bromide (MTT, 5 mg/ml) solution for 3 h, and the resultant formazan crystals were dissolved in 100 μL of dimethyl sulfoxide (DMSO). The absorbance was measured by a microreader at 570 nm. The results were expressed as a percentage of treated cell numbers relative to the untreated control cells.

### 4.6. UVB Irradiation and TNF-α Treatment

HaCaT cells were seeded (0.1 × 10^5^ cells/mL) in 6-well plates in DMEM medium with 10% FBS for 24 h. After stabilization, cells were pretreated with pure-inotodiol and inotodiol concentrate for 18 h. Cells were washed with PBS before irradiation, and after washing, 1 mL of PBS was added to each well. For UVB irradiation, PBS was removed, and DMEM without serum was added to each cell. Cells were incubated for 18 h. The total energy of irradiation was 10 mJ/cm^2^ (wavelength 312 nm, 10 s). For treatment with TNF-α, HaCaT cells were treated with 20 μg/mL TNF-α for 6 h at 37 °C. 

### 4.7. Quantitative Polymerase Chain Reaction (qPCR)

Total RNA was isolated from HaCaT cells using TRIzol (Life Technologies, Carlsbad, CA, USA) according to the manufacturer’s protocol and a previously described method with a slight modification [17]. cDNA was synthesized from 1 µg RNA by a reverse transcriptional reaction. The qPCR amplification was performed with SYBR-Green/ROX qPCR Master Mix (2×) (BioFact Co., Daejeon, Republic of Korea). The PCR primers for IL-6, TNF-α, IL-1β, iNOS, PLTP, APOE, ABCA1, and GAPDH were obtained from Bioneer Corp. Primer sequences are listed in Table 1.

### 4.8. Statistical Analyses

At least three independent experiments were performed in triplicate, and the results were presented as means ± standard deviation (SD). A statistical comparison between experimental groups and controls was performed using Student’s *t*-tests (Excel 2016, Microsoft Corp., Redmond, WA, USA). *p* values less than 0.05 were considered significant.

## 5. Conclusions

In conclusion, our results demonstrated that pure inotodiol and lanostane triterpenoids-rich inotodiol concentrate exert anti-inflammatory activities under various stimulations, such as stimulation with UV or TNF-α, which might prevent NF-κB signaling activation, but it should be further elucidated. Pure inotodiol/inotodiol concentrate could also modulate the synthesis of collagen and hyaluronic acid by controlling COL1A2 and HAS2/3 expression, which implies a crucial role for inotodiol/inotodiol concentrate in the prevention of skin aging. These results illuminate the anti-inflammatory and anti-aging effects of inotodiol/inotodiol concentrate, and it is highly conceivable that inotodiol/inotodiol concentrate is a promising natural bioactive substance to be incorporated in therapeutic and beautifying applications.

## Figures and Tables

**Table 1:** Genes and PCR primers used in this study.

Gene	Forward	Reverse
GAPDH	AAGTGGATATTGTTGCCATC	ACTGTGGTCATGAGTCCTTC
IL-6	ATGAACTCCTTCTCCACAAGC	GTTTTCTGCCAGTGCCTCTTTG
IL-8	TCTGTGTGAAGGTGCAGTT	AGCCCTCTTCAAAAACTTCT
IL-1β	AAACAGATGAAGTGCTCCTTCCAGG	TGGAGAACACCACTTGTTGCTCCA
TNF-α	GAGCTGAGAGATAACCAGCTGGTG	CAGATAGATGGGCTCATACCAGGG
HAS-2	TGGGTGTGTTCAGTGCAT	GCATTGTACAGCCATTCTCG
HAS-3	CCCAGCCAGATTTGTTGATG	AGTGGTCACGGGTTTCTTCC
COL1A1	AGCCAGCAGATCGAGAACAT	TCTTGTCCTTGGGGTTCTTG
COL1A2	TCAAGGTTTCCAAGGACCTG	GTGTCCCCTAATGCCTTTGA
HYAL-3	CAGTCCATTGGTGTGAGTG	CACAGGTGTAGAAAGGCTTC

## Data Availability

Not applicable.

## Supplementary Materials

The supporting information can be downloaded at: https://www.mdpi.com/article/10.3390/ijms241612803/s1.
