# Supplementary material for: Protective Effects of Lanostane Triterpenoids from Chaga Mushroom in Human Keratinocytes, HaCaT Cells, against Inflammatory and Oxidative Stresses
Source: Int J Mol Sci. 2023 Aug 15;24(16):12803. doi: 10.3390/ijms241612803 (PMC10454022; doi:10.3390/ijms241612803)
Supplement: Supplementary file 1 [file ijms-24-12803-s001.zip › ijms-2504029-supplementary.pdf]

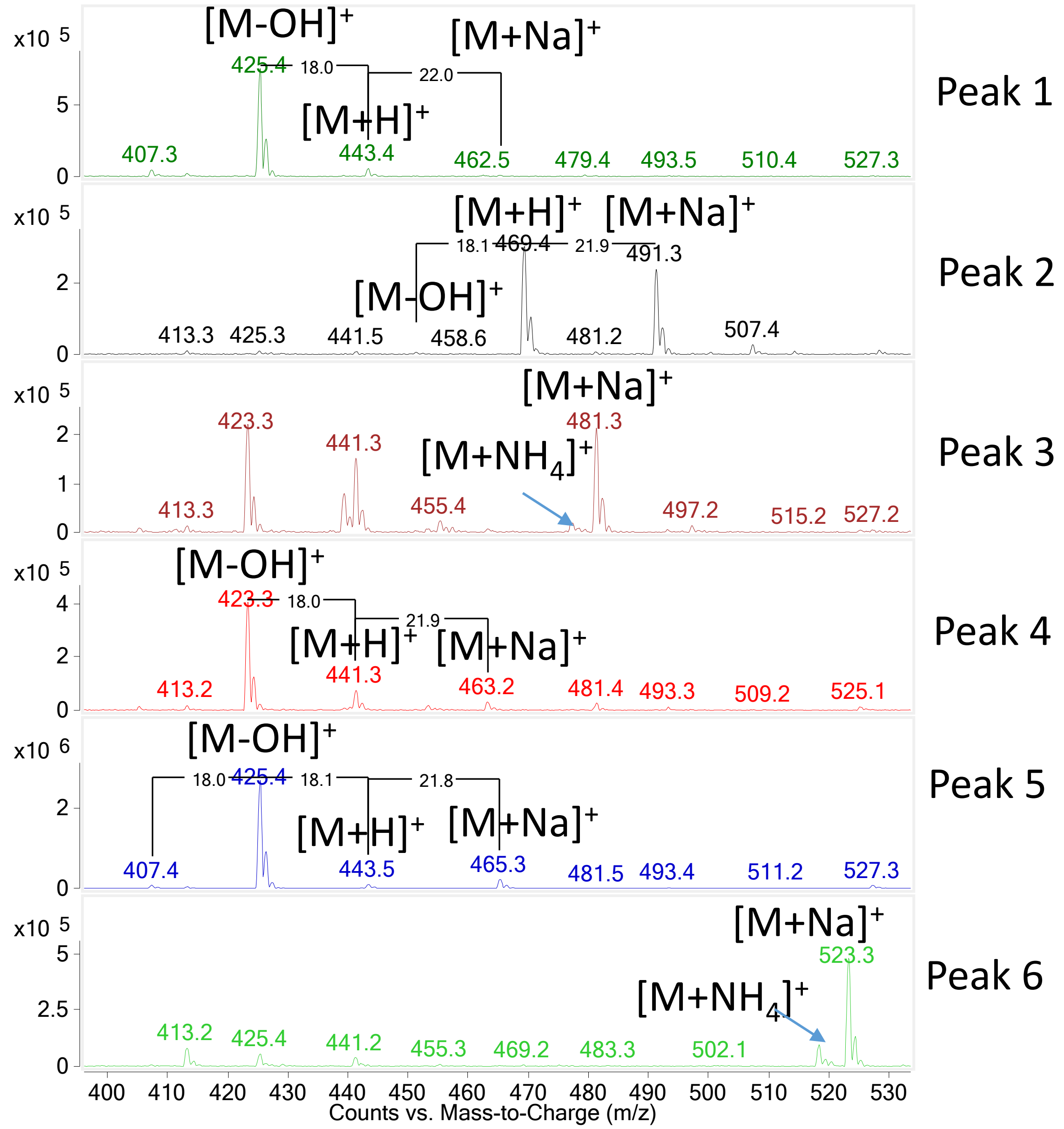

Supplementary figure 1. Mass spectra of major lanostane triterpenoids in Chaga mushroom extract
